# Job Satisfaction Among Healthcare Professionals During and Directly After the COVID-19 Pandemic: A Systematic Review of Psychosocial, Organizational and System-Level Factors

**DOI:** 10.3390/healthcare14213817

**Published:** 2026-11-09

**Authors:** Dawid Makowicz, Renata Dziubaszewska, Krzysztof Bryniarski, Katarzyna Lisowicz, Danuta Zarzycka

**Affiliations:** 1 Department of Health, State University of Applied Sciences in Krosno, Rynek 1, 38-400, Krosno, Poland; 2 Department of Interventional Cardiology, John Paul II Hospital, Institute of Cardiology, Jagiellonian University Medical College, Prądnicka 80, 31-202 Kraków, Poland; 3 Department of Pediatrics and Pediatric Nursing, Medical University of Lublin, Witolda Chodźki 7, 20-093 Lublin, Poland

**Keywords:** COVID-19, job satisfaction, physicians, nursing, paramedics

## Abstract

Background/Objectives: The COVID-19 pandemic substantially changed working conditions in healthcare and increased the workload of healthcare professionals. This systematic review aimed to identify factors associated with job satisfaction among physicians, nurses, and paramedics during the COVID-19 pandemic, with particular emphasis on psychosocial, organizational, and system-level factors. Methods: A systematic review was conducted in accordance with PRISMA guidelines. Searches were performed in ScienceDirect (10 January 2026), PubMed (16 January 2026), and Scopus (22 January 2026). Original studies published between 2020 and 2025 in English or Polish were eligible. Three researchers independently conducted the study selection and methodological assessment. Methodological quality was assessed using JBI critical appraisal tools. Fifteen studies involving physicians, nurses, and paramedics were included. Findings were synthesized narratively because of substantial heterogeneity among the included studies. The review was registered in the Open Science Framework. Conclusions: Higher job satisfaction was associated with older age, longer length of service and higher remuneration. In contrast, lower job satisfaction was associated with direct work with patients infected with SARS-CoV-2, higher levels of stress and burnout, and poor work organization. Limitations: The main limitations were related to the fact that only three databases were searched, the heterogeneity of the studies, and a language restriction, as only articles in Polish and English were included in the search.

## 1. Introduction

The COVID-19 pandemic represented one of the greatest challenges faced by healthcare systems in the 21st century, affecting both their functioning and the working conditions of healthcare professionals. The sudden increase in demand for healthcare services, constraints on the availability of human resources, and pre-existing workforce shortages increased the burden on healthcare professionals and exposed organizational limitations and difficulties in responding to crisis situations [1,2]. At the same time, the pandemic highlighted the importance of adequate workforce and organizational preparedness and the need to strengthen the resilience of healthcare systems to future epidemic threats [2,3]. Changes occurring within healthcare systems were directly reflected in the working conditions of healthcare professionals. The risk of infection, the need to use personal protective equipment, changes in working conditions, and an expanded scope of duties constituted sources of both physical and psychological burden [4,5]. This was particularly relevant to professions directly involved in patient care, including nurses, physicians, and paramedics [5,6,7,8,9]. An additional challenge was the need to adapt work organization to the dynamically changing epidemiological situation and resource availability, which further increased the demands placed on healthcare professionals [1,2].

The consequences of working under conditions of prolonged epidemic threat also affected the psychological well-being of healthcare professionals. Studies reported high levels of occupational stress, burnout, and deterioration in the quality of life of healthcare professionals [4,6]. Psychological burden may have resulted from the direct risk of infection and responsibility for patients’ health, as well as from an increased workload, organizational uncertainty, and the need to work under conditions of limited resources [4,5,6]. These phenomena were observed across different professional groups, with particular emphasis on nurses, physicians, and paramedics [5,6,7,8,9].

Under these circumstances, job satisfaction among healthcare professionals assumed particular importance as one dimension of employees’ functioning within the occupational environment. It is associated not only with individuals’ appraisal of their working conditions, but also with psychological well-being, occupational stress, burnout, and engagement in professional duties [5,6,7]. Research findings indicate that the COVID-19 pandemic was associated with changes in job satisfaction among healthcare professionals, with such relationships observed across all professional groups [5,6].

Job satisfaction is not associated with a single factor; rather, it is related to the interplay of psychosocial, organizational, and system-level factors. Among psychosocial factors, occupational stress, emotional burden, burnout, and psychological well-being are of particular importance [4,5,6]. Organizational factors include work organization, scope of duties, availability of staff and resources, occupational safety, and organizational support [4,5,7,8]. At the system level, relevant factors include shortages of healthcare personnel, resource availability, and the capacity of the healthcare system to respond to increased demand for healthcare services [1,2,10]. Workforce shortages are particularly important in this context, as they predated the pandemic and were further brought to light by it [1,2,10]. The epidemiological crisis exposed and intensified problems related to the functioning of the healthcare labor market, affecting staff availability and the organization of healthcare delivery [2,10]. These factors may also be associated with job satisfaction by shaping employees’ appraisal of the work environment, their ability to perform professional duties, and their well-being [1,5].

The significance of healthcare professionals’ working conditions therefore extends beyond the individual employee. Job satisfaction may be associated with work engagement, organizational functioning, and workforce stability [5,7,8]. Experience from the pandemic also indicates that strengthening healthcare-system resilience requires consideration of both organizational resources and arrangements, as well as the needs and working conditions of personnel [2,3]. Accounting for these elements is particularly important in the context of long-term changes in workforce functioning and the preparedness of healthcare systems for future crises [1,9].

Job satisfaction among healthcare professionals should therefore be regarded as a multidimensional phenomenon associated with individual well-being, team functioning, and organizational and system-level conditions. Analysis of its factors during and directly after the COVID-19 pandemic requires consideration of the interplay among psychosocial and organizational factors and factors related to the functioning of the healthcare system as a whole [5,6,7,9].

To the best of our knowledge, the present study is the first systematic review to examine job satisfaction concurrently across three distinct professional groups—physicians, nurses, and paramedics—while considering the psychosocial, organizational, and occupational factors associated with job satisfaction in the context of the COVID-19 pandemic.

The aim of this article was to systematically review the scientific evidence on factors associated with job satisfaction among physicians, nurses, and paramedics during and directly after the COVID-19 pandemic, with particular emphasis on psychosocial, organizational, and system-level factors.

## 2. Materials and Methods

### 2.1. Protocol and Registration

This systematic review was prepared in accordance with the PRISMA guidelines for reporting systematic reviews, using a structured search strategy and clearly defined study inclusion and exclusion criteria [11].

The protocol for this systematic review was registered retrospectively on the Open Science Framework (OSF) on 2 October 2026 (DOI: 10.17605/OSF.IO/M74UA). The registration took place after the literature searches had been completed. Therefore, the registration should be considered retrospective rather than prospective.

### 2.2. Eligibility Criteria

Studies were included if they met all predefined eligibility criteria. Original research studies published between 1 January 2020 and 31 December 2025, addressing job satisfaction among healthcare professionals in the context of the COVID-19 pandemic were considered. The study population comprised physicians, nurses, and paramedics.

Physicians, nurses, and paramedics were selected because of their direct involvement in patient care and frequent contact with patients with COVID-19; therefore, profession-specific terms were incorporated into the search strategy to identify relevant evidence for these three occupational groups.

Studies were eligible for analysis if job satisfaction constituted an outcome variable and was assessed using a measurement instrument or a clearly specified assessment method. Studies examining associations between the level of job satisfaction and psychosocial, organizational, occupational, or other factors related to the functioning of healthcare professionals during and directly after the COVID-19 pandemic were included.

Studies published in Polish or English for which the full text was available were included. Only original articles reporting primary research findings were eligible for the review.

Studies were excluded if findings on job satisfaction among physicians, nurses, or paramedics could not be separated from those pertaining to other professional groups.

### 2.3. Information Sources and Search Strategy

Data were collected through a systematic review of scientific articles. Searches were conducted in the electronic databases ScienceDirect, PubMed, and Scopus. To identify publications in these databases, search strategies based on the simultaneous combination of the following terms were used:

- for physicians: “COVID-19” AND “Job Satisfaction” AND “Physicians”;
- for nurses: “COVID-19” AND “Job Satisfaction” AND “Nursing”;
- for paramedics: “COVID-19” AND “Job Satisfaction” AND “Paramedic”.

The following search filters and limits were applied: publication years 2020–2025 in all databases; in ScienceDirect, article type was restricted to Research articles, language to English, and access type to Open access and Open archive; in PubMed, text availability was restricted to Full text and article language to English or Polish; and in Scopus, document type was restricted to Article and language to English.

The searches were completed on 10 January 2026 for ScienceDirect, 16 January 2026 for PubMed, and 22 January 2026 for Scopus.

### 2.4. Study Selection Process

Before the main search was conducted, a preliminary pilot search was performed to assess the sensitivity and adequacy of the planned search strategy and to identify potentially eligible publications. The initial strategy combined terms related to the COVID-19 pandemic and job satisfaction.

Analysis of the pilot-search results showed that the absence of specific terms referring to healthcare personnel led to the identification of a substantial number of publications concerning other occupational groups or populations that did not meet the inclusion criteria. Accordingly, before the main search and selection process began, the search strategy was refined by applying separate terms for the three predefined professional groups: physicians, nurses, and paramedics.

The databases were searched independently by three researchers (DM, RD, and KB) between 1 January and 22 January 2026. Each of the three researchers (DM, RD, and KB) performed data extraction independently and in duplicate; duplicates were excluded based on the article title, authors, and year of publication. Disagreements among the authors conducting the searches were resolved through discussion and/or consultation with the remaining authors (DZ and KL). In addition, the researchers met regularly to clarify questions and/or resolve inconsistencies.

Only original articles published between 2020 and 2025 were eligible for analysis. Review articles and case reports were excluded because they did not meet the predefined inclusion criteria.

### 2.5. Assessment of Completeness and Transparency of Reporting (CROSS)

The completeness and transparency of reporting in the included studies were assessed using the Consensus-Based Checklist for Reporting of Survey Studies (CROSS) (Supplementary Material) [12]. This checklist was used exclusively to evaluate the extent to which relevant information about the study design, methods, participants, data collection, and results was adequately reported. It was not used to assess methodological quality, internal validity, or risk of bias. Accordingly, CROSS scores were interpreted as indicators of reporting completeness and transparency and were considered separately from the methodological quality and risk-of-bias assessment performed using the Joanna Briggs Institute (JBI) critical appraisal tools. The assessment was performed independently by three researchers (DM, RD, and KB), with uncertainties discussed with the remaining authors (DZ and KL).

### 2.6. Assessment of Methodological Quality

To assess methodological quality and the risk of bias in studies included in the review, the Joanna Briggs Institute (JBI) critical appraisal tool appropriate to each study design was used. The assessment covered key methodological elements, including: Q1—Were the criteria for inclusion in the sample clearly defined? Q2—Were the study subjects and the setting described in detail? Q3—Was the exposure measured in a valid and reliable way? Q4—Were objective, standard criteria used for measurement of the condition? Q5—Were confounding factors identified? Q6—Were strategies to deal with confounding factors stated? Q7—Were the outcomes measured in a valid and reliable way? Q8—Was appropriate statistical analysis used? Each criterion was assessed in accordance with the instructions for the relevant JBI checklist and rated as “Yes,” “No,” “Unclear,” or “Not applicable,” depending on the available information and whether the criterion was met [13]. The assessment was performed independently by three researchers (DM, RD, and KB), with uncertainties discussed with the remaining authors (DZ and KL). This assessment was conducted independently of the CROSS reporting assessment, which evaluated reporting completeness and transparency rather than methodological quality or risk of bias.

### 2.7. Data Synthesis

Because the included studies differed in their populations, outcome measures, and reporting of statistical results, and because comparable effect estimates were not consistently available, the findings were synthesized narratively rather than quantitatively. Results were organized according to the predefined domains of psychosocial, organizational, and system-level factors. Within these domains, the direction and consistency of reported associations were considered, while differences in measurement and study context were retained in the interpretation. Statistical significance alone was not treated as evidence of a stronger association, and findings from studies using different instruments were not interpreted as directly comparable in magnitude. No pooled effect estimates were calculated.

PSYCHOSOCIAL FACTORS

Stress was classified as a psychosocial factor because it reflects the psychological strain experienced by healthcare professionals in response to occupational demands. The included studies examined its relationship with employees’ subjective assessment of job satisfaction.

Burnout was included in the psychosocial domain because it reflects work-related psychological exhaustion and difficulties associated with prolonged occupational demands. Its relationship with job satisfaction concerns employees’ psychological well-being and experience of their professional roles.

Gender was included in this domain to examine whether job satisfaction differed between demographic groups.

Age and professional experience were considered in this domain because they may be associated with individual adaptation to occupational demands, accumulated experience, and perceptions of work.

Individual factors were grouped together because they relate to personal health, social circumstances, and individual competencies that may be associated with job satisfaction. The included studies examined, among other factors, self-rated health, living arrangements, and communication effectiveness when interacting with patients with COVID-19.

ORGANIZATIONAL FACTORS

Occupational position and work organization were classified as organizational factors because they relate to employees’ roles, responsibilities, and the way work is structured within a healthcare workplace. Managerial position and work organization may influence employees’ experience of their duties and their assessment of job satisfaction.

Remuneration was classified as an organizational factor because financial compensation is an important aspect of employment conditions and may influence employees’ evaluation of their work.

SYSTEM-LEVEL FACTORS

Profession was included in this domain to compare job satisfaction across physicians, nurses, and paramedics, whose responsibilities and working conditions may differ.

Contact with patients infected with SARS-CoV-2 was considered in the context of the pandemic’s impact on healthcare delivery and the distribution of occupational exposure. More precisely, it represents an occupational exposure factor that may be shaped by professional responsibilities and workplace arrangements, rather than a system-level factor in itself.

## 3. Results

### 3.1. Study Selection

The searches of ScienceDirect, PubMed, and Scopus using the specified keywords yielded 2267 records: 954 from ScienceDirect (374 concerning physicians, 533 nursing personnel, and 47 paramedics), 641 from PubMed (136 concerning physicians, 501 nursing personnel, and 4 paramedics), and 672 from Scopus (236 concerning physicians, 424 nursing personnel, and 12 paramedics). A total of 868 duplicate records were removed, leaving 1399 records for further analysis.

**Figure 1:**
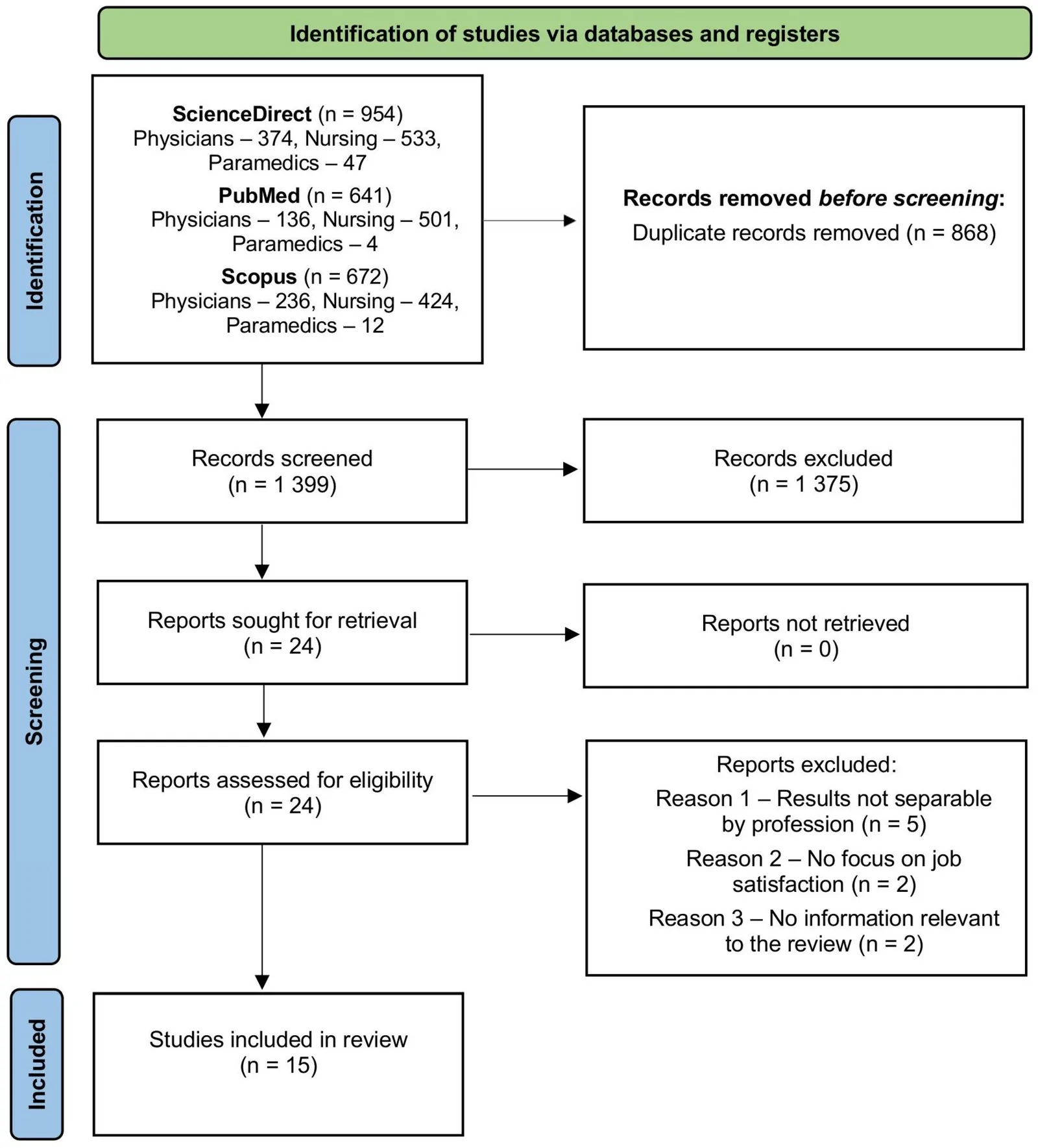
Study selection process (PRISMA).

After titles and abstracts were screened against the inclusion criteria, 1375 records were excluded. Twenty-four articles underwent further assessment, all of which were available in full text in English. Following full-text review, nine articles were excluded for the following reasons: inability to separate results by individual healthcare profession (n = 5), lack of a focus on job satisfaction (n = 2), and lack of information relevant to the review (n = 2). The process of record and article selection according to the PRISMA flow diagram is presented in Figure 1.

### 3.2. Characteristics of Included Studies

Fifteen studies on job satisfaction among healthcare professionals during and directly after the COVID-19 pandemic were included in the systematic review. The studies involved physicians, nurses, and paramedics and were conducted in Poland, Iran, Greece, Jordan, Italy, Germany, Argentina, South Korea, Brazil, and Israel. Sample sizes ranged from 77 to 7681 participants. Because of substantial heterogeneity across studies, including differences in populations, professional groups, healthcare systems, study periods, instruments used to measure job satisfaction, definitions of the factors analyzed, and methods of reporting results, quantitative pooling of findings in a single meta-analysis was not considered appropriate. The possibility of conducting separate meta-analyses within more homogeneous subsets of studies, including specific professional groups, was also considered. However, this approach was not feasible because substantial methodological and measurement-related heterogeneity remained even within these subsets. In particular, studies differed in the instruments used to assess job satisfaction, scoring systems, outcome definitions, and the statistical data reported. Consequently, no subsets of studies were considered sufficiently comparable to support reliable quantitative pooling. The findings were therefore synthesized narratively and grouped into three main domains: psychosocial, organizational, and system-level factors. This approach is appropriate when study characteristics do not permit a reliable pooled effect estimate. The instruments used to assess job satisfaction varied considerably across the included studies. These included standardized instruments, such as the Minnesota Satisfaction Questionnaire (MSQ), the Job Satisfaction Scale (JSS), the Short Index of Job Satisfaction (SIJS), and the Brief Job Satisfaction Index (BJSS), as well as study-specific questionnaires and modified instruments. Differences were observed in the number of items, response formats, scoring ranges, and classification thresholds used to interpret satisfaction levels. For example, although some studies used the short version of the MSQ with the same total score range (20–100), different cut-off values were applied to classify low, moderate, and high job satisfaction. Similarly, instruments referred to as the JSS differed in their structure and scoring systems. Higher scores generally indicated greater job satisfaction; however, the numerical scores and categorical interpretations were not directly comparable across all studies. These differences were taken into account when synthesizing the findings narratively.

### 3.3. Methodological Quality of Included Studies

Not all included studies focused exclusively on the selected healthcare professions. For studies involving other occupational groups, a detailed review of the reported findings was conducted, and only information pertaining to physicians, nurses, and paramedics was extracted.

Methodological quality assessment using the JBI tools indicated generally satisfactory study quality. One study received a score of 8/8, ten studies received 7/8, and four studies received 6/8. Overall, the studies demonstrated good methodological quality; however, in most studies, unclear reporting of strategies for addressing confounding factors remained a limitation (Table 1).

The numerical scores should not be interpreted as validated overall measures of methodological quality, particularly when individual risk-of-bias domains remain unclear.

The characteristics of the studies included in the systematic review, including authors, title, year of publication, study sample, variables examined, instruments used to assess job satisfaction, and country of study, together with the assessment of reporting completeness using the CROSS checklist, are presented in Table 2.

## 4. Discussion

### 4.1. Psychosocial Factors

#### 4.1.1. Stress

Across all professional groups in which this relationship was assessed, higher levels of perceived occupational stress were associated with lower job satisfaction. This association was observed among physicians [18], nurses [15], and paramedics [26]. These findings indicate a consistent relationship between the psychological burden associated with working during the pandemic and the appraisal of job satisfaction.

#### 4.1.2. Burnout

Burnout was among the factors associated with lower job satisfaction. In the studies analyzed, lower levels of burnout were associated with higher job satisfaction [14,24,26]. At the same time, lower job satisfaction was observed among employees who reported an intention to leave their profession [28]. In one of the analyzed studies, the greatest propensity to change jobs was observed among nursing personnel, whereas the lowest was observed among physicians [16]. These findings indicate that job satisfaction co-occurred with work-related burden and stated intentions regarding continued employment; however, the direction of causality cannot be determined from the studies analyzed.

#### 4.1.3. Gender

The association between gender and job satisfaction was inconsistent. In some studies, women reported higher levels of job satisfaction [19], whereas another study found lower levels of satisfaction among women [21]. Other studies found no significant association between gender and job satisfaction [23,27]. Thus, the evidence analyzed did not provide a consistent basis for identifying gender as a clear factor differentiating levels of job satisfaction. These inconsistent findings may reflect differences in occupational roles, working conditions, and organizational contexts across the included studies, suggesting that the association between gender and job satisfaction should be interpreted in relation to the broader work environment.

#### 4.1.4. Age and Professional Experience

Older age [14,15] and longer length of service [17,21] were associated with higher self-reported job satisfaction. Although career adaptability and openness toward organizational change have been examined in the broader organizational literature [29,30], these constructs were not directly assessed in the studies included in the present review. Therefore, no conclusions can be drawn regarding whether longer professional experience improves healthcare professionals’ ability to adapt to organizational change. However, this association should be interpreted cautiously, as it may be partly affected by self-selection bias. Healthcare professionals who are dissatisfied with their jobs may be more likely to leave their workplace or the profession, meaning that those with longer tenure may not be representative of all individuals who initially entered the profession. Recent research has documented associations between job dissatisfaction and turnover, supporting the need to consider this potential source of bias when interpreting the relationship between age, length of service, and job satisfaction [31,32].

#### 4.1.5. Individual Factors

Higher self-rated current health status was associated with higher job satisfaction [15,22]. A similar association was observed for living with other people compared with living alone [17]. In addition, greater effectiveness in communicating with patients with COVID-19 was associated with higher job satisfaction [23]. These findings therefore indicate that job satisfaction was associated not only with characteristics of the work environment itself, but also with individuals’ appraisal of their health status, social circumstances, and communication competencies. These individual-level factors may also interact with organizational conditions, as supportive working environments may help healthcare professionals manage work-related demands and maintain their well-being, although such interactions were not directly established by the studies included in this review.

### 4.2. Organizational Factors

#### 4.2.1. Occupational Position and Work Organization

The nature of the position held and conditions of work organization were associated with job satisfaction. Holding a managerial position was associated with higher self-reported job satisfaction [27,28]. The employment setting was also relevant: community nurses reported higher job satisfaction than nurses working in hospital wards [28]. By contrast, staffing shortages [21] and limited availability of personal protective equipment [26,28] were associated with lower job satisfaction. Of particular note, only 17.6% of participants reported being satisfied with work organization during the pandemic [20]. These findings suggest that organizational conditions may be relevant not only to job satisfaction directly but also to healthcare professionals’ psychological well-being, particularly when staffing shortages and limited protective equipment increase the demands associated with patient care.

#### 4.2.2. Remuneration

Remuneration was another recurrent factor associated with job satisfaction. Lower income was associated with lower satisfaction [14], whereas higher income was positively associated with self-reported job satisfaction [17,19]. In a study assessing individual components of job satisfaction, the greatest decline concerned satisfaction with remuneration [20]. Furthermore, a reduction in income during and directly after the pandemic was associated with lower job satisfaction [22]. Thus, the association between financial circumstances and job satisfaction was consistent across the analyzed material. The relationship between remuneration and job satisfaction has also been confirmed in studies conducted in sectors other than healthcare [33,34,35].

### 4.3. System-Level Factors

#### 4.3.1. Profession

Job satisfaction differed among physicians, nurses, and paramedics; however, the direction of these differences was not consistent across studies [16,17,20,21]. Therefore, on the basis of the analyzed evidence, membership in a particular profession alone cannot be regarded as an unequivocal factor associated with job satisfaction. Differences among professional groups should be interpreted in the context of differences in working conditions, scope of duties, and organization of healthcare delivery.

A clear imbalance in the number of studies addressing the different professional groups was evident in the analyzed material. Studies involving nurses and physicians constituted the vast majority of the evidence, whereas substantially fewer studies addressed paramedics. The limited representation of paramedics restricts the ability to draw firm conclusions regarding this group and to make direct comparisons between their job satisfaction and that of physicians and nurses.

#### 4.3.2. Occupational Exposure to Patients with COVID-19

Fear of COVID-19 was associated with lower job satisfaction among physicians [18]. Among Polish nurses, fear of becoming infected with SARS-CoV-2 was frequently reported as a work-related concern; however, coronavirus anxiety was not significantly associated with job satisfaction [15]. Experience of working with patients infected with SARS-CoV-2 was likewise associated with a more negative appraisal of job satisfaction [19,28]. It is also noteworthy that healthcare professionals employed in non-isolation hospitals reported higher job satisfaction [19]. Home quarantine was associated with higher levels of perceived anxiety and poorer self-rated health [15]. Healthcare professionals with experience of working with patients with COVID-19 also more frequently reported an intention to leave their jobs [25]. Taken together, these findings indicate that contact with patients with COVID-19 was associated not only with lower job satisfaction but also with anxiety, poorer self-rated health, and a greater intention to leave employment.

#### 4.3.3. Overall Pattern of Findings

The overall pattern of findings indicates the most consistent associations between job satisfaction and psychosocial burden, work-organization conditions, economic circumstances, and exposure to COVID-19-related risks. Higher levels of stress and burnout, staffing shortages, limited availability of personal protective equipment, less favorable financial circumstances, and contact with patients infected with SARS-CoV-2 were associated with lower job satisfaction. By contrast, higher remuneration, greater professional experience, holding managerial positions, better self-rated health, and greater effectiveness in communicating with patients were associated with higher job satisfaction. An important aspect of the findings is the distinction between the acute phase of the COVID-19 pandemic and the subsequent period. Studies conducted during the pandemic highlighted the relevance of immediate psychosocial and occupational burdens. For example, among Polish physicians, higher perceived stress and greater fear of COVID-19 were associated with lower job satisfaction [18]. Similarly, nurses caring for patients with confirmed COVID-19 and those reporting insufficient availability of personal protective equipment had lower job satisfaction [28]. The relationship between burnout and job satisfaction was also documented among nurses working in COVID-19 wards [24]. However, these associations were not consistent across all studies. For instance, coronavirus-related anxiety was not found to be a statistically significant predictor of job satisfaction among Polish nurses [15].

Evidence from the period immediately following the first pandemic wave also points to the importance of working conditions. In their study of more than 7000 healthcare professionals in Italy, Barili et al. [16] found that changes in working conditions associated with the health emergency were significantly related to lower job satisfaction. The authors also emphasized the importance of organizational and contextual factors, including the quality of healthcare facilities and perceived shortages of medical personnel.

Importantly, the end of the acute phase did not necessarily coincide with the resolution of occupational and psychosocial difficulties. A study conducted in June 2023 by Galanis et al. [21] reported high levels of burnout and lower job satisfaction among nurses compared with other healthcare professionals. These findings suggest that difficulties related to work organization and the psychological burden experienced by healthcare professionals may persist beyond the period of the most intense epidemiological threat.

Nevertheless, these observations should be interpreted cautiously. The included studies were not designed to provide direct longitudinal comparisons between the acute phase of the pandemic and the subsequent period. Furthermore, differences in study populations, measurement instruments, and healthcare contexts limit the comparability of the findings. Therefore, the available evidence allows the identification of factors reported in different periods of the pandemic but does not establish a definitive temporal change in their relationship with job satisfaction.

#### 4.3.4. Strengths of the Review

This systematic review covers the COVID-19 pandemic period from 2020 to 2025, allowing both its early and later phases and the changing working conditions of healthcare professionals to be captured. The use of a structured search procedure based on the PRISMA guidelines, with clearly defined inclusion and exclusion criteria, together with the involvement of three independent researchers in the record-selection process, increases the transparency and credibility of the review process. An additional strength is the explicit focus on three professional groups—physicians, nurses, and paramedics—which enables a more precise analysis of factors associated with job satisfaction within individual professions.

### 4.4. Limitations

This review also has several limitations that should be considered. Only articles published in English or Polish and available in full text were included, which may have resulted in the omission of studies published in other languages or in subscription-based journals. Searches were conducted in only three databases (ScienceDirect, PubMed, and Scopus), which, despite the structured search strategy and involvement of several researchers in study selection, does not guarantee complete identification of all studies meeting the eligibility criteria. Only original articles were included in the analysis, which strengthened the empirical basis of the conclusions but may have narrowed the perspective by excluding evidence from secondary reviews.

An important limitation is the heterogeneity of the instruments used to measure job satisfaction. Furthermore, differences in scoring systems and cut-off values used to classify job satisfaction levels, including among studies using the same instrument, limited direct comparisons of absolute scores and satisfaction categories. Consequently, the synthesis focused primarily on the direction and consistency of reported associations rather than on comparing absolute job satisfaction levels across studies. Differences among countries, healthcare systems, and stages of the COVID-19 pandemic during which the studies were conducted may further limit the generalizability of the findings to other contexts. Furthermore, the included studies varied in the statistical measures used and reported, and effect estimates were not consistently available across all studies. Consequently, the magnitude of associations between job satisfaction and the examined factors could not be compared systematically across all included studies. The findings should therefore be interpreted primarily in terms of the direction and consistency of the reported associations rather than their relative magnitude. The access filters applied in ScienceDirect and PubMed may have resulted in the omission of some potentially eligible publications that were not available in full text or through open-access sources. In addition, grey literature was not searched.

## 5. Conclusions

Job satisfaction among healthcare professionals during and directly after the COVID-19 pandemic was multifactorial and associated with psychosocial, organizational, occupational, and individual factors. The most consistent associations with lower job satisfaction concerned higher levels of occupational stress and burnout, staffing shortages, limited availability of personal protective equipment, less favorable financial circumstances, and direct contact with patients infected with SARS-CoV-2. In contrast, higher remuneration, longer professional experience, holding managerial positions, better self-rated health, and greater effectiveness in communicating with patients were associated with higher job satisfaction. These findings highlight the importance of considering both the psychosocial well-being of healthcare professionals and the organizational conditions in which they provide care.

Because most included studies were observational, the identified associations do not establish causal relationships, and the extent to which modifying these factors would improve job satisfaction remains uncertain. Nevertheless, the findings identify potential areas for attention in healthcare workforce management, including adequate staffing, access to personal protective equipment, appropriate remuneration, organizational support, and the prevention of occupational stress and burnout. The effectiveness of specific interventions should be evaluated in prospective longitudinal and intervention-based studies.

### Implications for Practice and Healthcare Systems

The findings of this review identify several factors associated with job satisfaction among healthcare professionals during and directly after the COVID-19 pandemic, including occupational stress and burnout, work organization, remuneration, and direct contact with patients infected with SARS-CoV-2. These associations highlight areas that may warrant attention from healthcare managers and policymakers. Potential priorities include reviewing staffing adequacy, ensuring the availability of personal protective equipment, strengthening organizational support, monitoring psychosocial burden, and evaluating approaches to stress and burnout prevention.

However, given the predominantly observational nature of the included evidence, these findings should not be interpreted as demonstrating that interventions targeting these factors will necessarily improve job satisfaction. Rather, they provide an evidence-informed basis for identifying potential areas for organizational and policy action. The effectiveness of specific measures should be evaluated prospectively, preferably through longitudinal or intervention-based studies using clearly defined outcomes.

At the health-system level, workforce planning, resource allocation, and remuneration policies may warrant further evaluation in relation to healthcare professionals’ job satisfaction and well-being. Such measures should be regarded as potential priorities for policy development rather than interventions with established causal effects on job satisfaction. Further research is needed to determine which organizational and system-level strategies are effective, for whom, and under what working conditions.

## Figures and Tables

**Table 1:** Assessment of methodological quality and risk of bias in the included studies using the JBI critical appraisal tools.

No.	Study	Q1	Q2	Q3	Q4	Q5	Q6	Q7	Q8	JBI Score
1	Alrawashdeh et al., 2021 [14]	Y	Y	Y	Y	Y	U	Y	Y	7/8
2	Andruszkiewicz et al., 2023 [15]	Y	Y	Y	Y	Y	U	Y	Y	7/8
3	Barili et al., 2022 [16]	Y	Y	Y	Y	Y	Y	Y	Y	8/8
4	Diakos et al., 2022 [17]	Y	Y	U	Y	U	U	Y	Y	6/8
5	Dymecka et al., 2021 [18]	Y	Y	Y	Y	Y	U	Y	Y	7/8
6	Dziedzic et al., 2023 [19]	Y	Y	Y	Y	Y	U	Y	Y	7/8
7	Eiche et al., 2021 [20]	Y	Y	Y	Y	Y	U	Y	Y	7/8
8	Galanis et al., 2023 [21]	Y	Y	Y	Y	Y	U	Y	Y	7/8
9	Galvalisi et al., 2022 [22]	Y	Y	Y	Y	U	U	Y	Y	6/8
10	Ghahremani et al., 2024 [23]	Y	Y	Y	Y	U	U	Y	Y	6/8
11	Heidari et al., 2022 [24]	Y	Y	Y	Y	Y	U	Y	Y	7/8
12	Kim et al., 2022 [25]	Y	Y	Y	Y	Y	U	Y	Y	7/8
13	Nassehi et al., 2023 [26]	Y	Y	Y	Y	U	U	Y	Y	6/8
14	Pirino et al., 2023 [27]	Y	Y	Y	Y	Y	U	Y	Y	7/8
15	Savitsky et al., 2021 [28]	Y	Y	Y	Y	Y	U	Y	Y	7/8

JBI criteria: Q1—Were the criteria for inclusion in the sample clearly defined? Q2—Were the study subjects and the setting described in detail? Q3—Was the exposure measured in a valid and reliable way? Q4—Were objective, standard criteria used for measurement of the condition? Q5—Were confounding factors identified? Q6—Were strategies to deal with confounding factors stated? Q7—Were the outcomes measured in a valid and reliable way? Q8—Was appropriate statistical analysis used? Legend: Y—Yes, N—No, U—Unclear.

**Table 2:** Characteristics and qualitative analysis of the studies included in the systematic review.

No.	Authors; Title; Year	Study Characteristics (Sample, Variables, Instruments for Measuring Job Satisfaction, Country of Study)	Results
1	Alrawashdeh HM, Al-Tammemi AB, Alzawahreh MK, Al-Tamimi A, Elkholy M, Al Sarireh F, Abusamak M, Elehamer NMK, Malkawi A, Al-Dolat W, Abu-Ismail L, Al-Far A, Ghoul I; Occupational burnout and job satisfaction among physicians in times of COVID-19 crisis: a convergent parallel mixed-method study; 2021 [14]	Study sample: 973 physicians. Variables associated with job satisfaction: gender, age, burnout level, income. Job satisfaction instrument: Short Index of Job Satisfaction (SIJS), consisting of 5 items rated on a 5-point scale (maximum score: 25); higher scores indicate greater job satisfaction. Country: Jordan.	The mean job satisfaction score was 15.3 (SD = 3.98) among male physicians and 15.5 (SD = 3.80) among female physicians. The difference in job satisfaction scores by gender was not statistically significant (*p* = 0.562). Older age was significantly associated with higher job satisfaction (*p* = 0.017). Predictors of lower job satisfaction included burnout (*p* < 0.001) and lower income (*p* = 0.008). CROSS: 35/40.
2	Andruszkiewicz A, Kruczek A, Betke K, Adamczyk K; Work anxiety factors, coronavirus anxiety and job satisfaction of Polish nurses facing COVID-19 pandemic outbreak; 2023 [15]	Study sample: 433 nurses. Variables associated with job satisfaction: stress at work, age, quarantine, current health status. Job satisfaction instrument: Satisfaction with Job Scale (SSP), consisting of 5 items rated on a 7-point scale (maximum score: 35); higher scores indicate greater job satisfaction. Country: Poland.	The mean job satisfaction score was 19.9 (SD = 7.8). Lower levels of occupational stress during the COVID-19 pandemic were associated with higher job satisfaction. Fear of becoming infected with SARS-CoV-2 was the most frequently reported work-related concern (25.4%). Coronavirus anxiety was not significantly associated with job satisfaction (*p* = 0.942). Older respondents reported higher job satisfaction (*p* < 0.001). Nurses who underwent home quarantine reported higher anxiety levels and poorer self-rated health. Higher self-rated current health status was associated with higher job satisfaction. CROSS: 36/40.
3	Barili E, Bertoli P, Grembi V, Rattini V; Job satisfaction among healthcare professionals in the aftermath of the COVID-19 pandemic; 2022 [16]	Study sample: 7681 respondents (2549 physicians, 4561 nurses, 571 other healthcare professionals). Variables associated with job satisfaction: profession, intention to change profession. Job satisfaction instrument: Study-specific instrument comprising 50 items rated on an 8-point scale; the mean score was calculated, with higher scores indicating greater job satisfaction. Country: Italy.	Mean job satisfaction scores were approximately 5.5 for physicians, 5.1 for nurses, and 5.8 for other healthcare professionals. The reported career change was 3.5 for physicians, 4.5 for nursing staff, and 4.0 for other healthcare professionals. CROSS: 35/40.
4	Diakos GE, Koupidis S, Dounias G.; Measurement of job satisfaction among healthcare professionals during the COVID-19 pandemic: A cross-sectional study; 2022 [17]	Study sample: 34 physicians, 33 nurses, 10 paramedics. Variables associated with job satisfaction: living arrangement, length of service, occupation, salary. Job satisfaction instrument: Job Satisfaction Scale (JSS), consisting of 36 items, with a total score ranging from 36 to 216 points; higher scores indicate greater satisfaction. Country: Greece.	The mean overall job satisfaction score among healthcare professionals was relatively low at 120.6 (SD = 25.58). Physicians reported the lowest level of satisfaction (112.8 ± 25.8), while nurses reported a score of 119 (SD = 24.7). Paramedics reported the highest level of satisfaction (134.7 ± 17.2). Healthcare professionals living with others were more satisfied with their jobs than those living alone (*p* < 0.05). Healthcare professionals with more than 20 years of experience had the highest overall job satisfaction (129.27, *p* < 0.05). Respondents receiving higher salaries also reported greater job satisfaction (*p* < 0.05). CROSS: 36/40.
5	Dymecka J, Filipkowski J, Machnik-Czerwik A.; Fear of COVID-19: stress and job satisfaction among Polish doctors during the pandemic; 2021 [18]	Study sample: 97 physicians. Variables associated with job satisfaction: fear of COVID-19, stress. Job satisfaction instrument: The Brief Job Satisfaction Index (BJSS), consisting of 5 items rated on a 5-point scale (maximum score: 25); higher scores indicate greater job satisfaction. Country: Poland.	The mean job satisfaction score among surveyed physicians was 19.31. Fear of COVID-19 was correlated with job satisfaction (*p* = 0.035) and perceived stress (*p* < 0.001). The relationship between job satisfaction and perceived stress was also statistically significant (*p* < 0.001). CROSS: 33/40.
6	Dziedzic B, Jagiełło M, Kobos E, Sienkiewicz Z, Czyżewski Ł; Job satisfaction among nurses working in hospitals during the COVID-19 pandemic; 2023 [19]	Study sample: 302 nurses. Variables associated with job satisfaction: gender, income, contact with a patient infected with SARS-CoV-2, and work in a non-isolated hospital. Job satisfaction instrument: Minnesota Job Satisfaction Questionnaire (MSQ)—short version, consisting of 20 items rated on a 5-point scale (maximum score: 100). Scores of 20–47, 48–76, and 77–100 indicate low, moderate, and high job satisfaction, respectively. Country: Poland.	Respondents achieved a mean job satisfaction score of 58.53 (SD = 11.42). Women reported significantly higher job satisfaction (*p* = 0.005). Furthermore, higher job satisfaction was observed among those employed in non-isolation hospitals (*p* = 0.041), with a higher monthly income (*p* = 0.001), and those not in contact with patients infected with SARS-CoV-2 (*p* = 0.005). CROSS: 32/40.
7	Eiche C, Birkholz T, Konrad F, Golditz T, Keunecke JG, Prottengeier J; Job Satisfaction and Performance Orientation of Paramedics in German Emergency Medical Services-A Nationwide Survey; 2021 [20]	Study sample: 2590 paramedics, control group: over 2000 healthcare professionals. Variables associated with job satisfaction: COVID-19 pandemic, income, satisfaction with work organization. Job satisfaction instrument: Neuberger and Allerbeck’s Job Satisfaction Questionnaire, consisting of 7 subscales, 79 items scored on a 1–4 scale; mean scores indicate the level of satisfaction (1 = low; 4 = high). Country: Germany.	The following scores were obtained across the 7 subscales: my activity—3.11; my colleagues—2.99; my supervisor—2.83; my development—2.56; my working conditions—2.57; organization and management—2.26; and my payment—2.09. A statistically significant decrease in job satisfaction (*p* < 0.01) was observed across all 7 subscales compared to the control group. The largest decrease was observed for the salary subscale (−0.64). Overall, 17.6% of respondents were satisfied with their work organization. CROSS: 38/40.
8	Galanis P, Moisoglou I, Katsiroumpa A, Vraka I, Siskou O, Konstantakopoulou O, Meimeti E, Kaitelidou D; Increased Job Burnout and Reduced Job Satisfaction for Nurses Compared to Other Healthcare professionals after the COVID-19 Pandemic; 2023 [21]	Study sample: 1760 healthcare professionals (including 946 nurses). Variables associated with job satisfaction: gender, occupation, staffing shortages, clinical experience, and employment sector. Job satisfaction instrument: Job Satisfaction Scale (JSS), consisting of 36 items, with scores ranging from 36 to 216; higher scores indicate greater satisfaction. Country: Greece.	The mean satisfaction score was 108.2 (SD = 30.9). Female gender (*p* = 0.001), nursing profession (*p* < 0.001), staff shortages (*p* < 0.001), and less clinical experience (*p* = 0.002) were significantly associated with lower job satisfaction scores. Employment sector was also significantly associated with perceived job satisfaction (*p* < 0.001). CROSS: 36/40.
9	Galvalisi N, Penalba M, Pérez A, Cabrera N, Mejía R; Emergency department physicians’ job satisfaction in the Argentine Republic during the COVID-19 pandemic; 2022 [22]	Study sample: 187 physicians. Variables associated with job satisfaction: income, self-rated health. Job satisfaction instrument: Minnesota Satisfaction Questionnaire (MSQ), consisting of 20 items rated on a 5-point scale (maximum score: 100). Scores of 20–50, 51–70, and 71–100 indicate low, medium, and high job satisfaction, respectively. Country: Argentina.	The surveyed physicians had a mean overall job satisfaction score of 58/100, with the highest score being 72 and the lowest being 47. Among the respondents, 27% had a high level of job satisfaction, while 32% had a low level. A reduction in income during the pandemic was significantly associated with lower job satisfaction (*p* = 0.012). Self-rated health was also associated with job satisfaction (*p* = 0.034). CROSS: 34/40.
10	Ghahremani L, Khoramaki Z, Kaveh MH, Karimi M, Nazari M, Orgambídez Ramos A; Communication Self-Efficacy and Job Satisfaction among Nurses during the COVID-19 Pandemic; 2024 [23]	Study sample: 364 nurses. Variables associated with job satisfaction: effectiveness in communicating with COVID-19 patients, gender. Job satisfaction instrument: A study-specific questionnaire consisting of 8 items rated on a 6-point scale (maximum score: 48); higher scores indicate greater job satisfaction. Country: Iran.	The mean job satisfaction score was 28.11 (SD = 6.48). Effectiveness in communicating with patients with COVID-19 was positively correlated with job satisfaction (*p* < 0.001). No statistically significant association between gender and job satisfaction was observed. CROSS: 36/40.
11	Heidari S, Parizad N, Goli R, Mam-Qaderi M, Hassanpour A; Job satisfaction and its relationship with burnout among nurses working in COVID-19 wards: A descriptive correlational study; 2022 [24]	Study sample: 251 nurses. Variables associated with job satisfaction: Burnout dimensions (emotional exhaustion, depersonalization, personal accomplishment). Job satisfaction instrument: Minnesota Satisfaction Questionnaire (MSQ)—short version, consisting of 20 items rated on a 5-point scale (maximum score: 100). Scores of 20–47, 48–76, and 77–100 indicate low, moderate, and high job satisfaction, respectively. Country: Iran.	The results showed that 75.7% of nurses had low job satisfaction, 12.7% had moderate job satisfaction, and 11.6% had high job satisfaction. Based on the correlation coefficient, the dimensions of professional exhaustion (r = −0.394, *p* < 0.001) and personal accomplishment (r = −0.590, *p* < 0.001) were inversely related to nurses’ job satisfaction. CROSS: 36/40.
12	Kim B, Kwon KT, Hwang S, Ryoo HW, Chung US, Lee SH, Lee JY, Park HY, Shin JY, Bae SG; Psychological Effects of COVID-19 Patient Management Experience among Paramedics and Emergency Medical Technicians: A Nationwide Survey in Korea; 2022 [25]	Study sample: 326 paramedics. Variables associated with job satisfaction: experience working with COVID-19 patients, intention to leave work. Job satisfaction instrument: A study-specific questionnaire categorizing respondents as dissatisfied, moderately satisfied, or satisfied with their jobs. Country: South Korea.	Of the respondents, 11.3% were dissatisfied with their jobs, 39.0% were moderately satisfied, and 49.7% were satisfied. A higher percentage of respondents with experience working with COVID-19 patients expressed job dissatisfaction, but the difference was not statistically significant (*p* = 0.327). However, the proportion of paramedics reporting willingness to leave their job if given the opportunity was higher in the group with experience working with COVID-19 patients (*p* < 0.001). CROSS: 35/40.
13	Nassehi A, Jafari J, Jafari-Oori M, Jafari M; Moral Distress, Burnout, and Job Satisfaction in Emergency Medical Technicians during the COVID-19 Pandemic; 2023 [26]	Study sample: 134 paramedics. Variables associated with job satisfaction: moral distress, burnout, limited access to personal protective equipment. Job satisfaction instrument: 5-point Likert scale (0–4); higher scores indicate greater job satisfaction. Country: Iran.	The mean job satisfaction score among paramedics during the COVID-19 pandemic was 3.63, which indicated a high level of job satisfaction. Higher moral distress was associated with lower job satisfaction, whereas lower levels of burnout were associated with higher job satisfaction. Both associations were statistically significant at *p* < 0.05. Paramedics with sufficient access to personal protective equipment reported higher job satisfaction than those with limited access to personal protective equipment (*p* < 0.05). CROSS: 35/40.
14	Pirino MVB, Nascimento Sobrinho CL, Dini AP; Professional satisfaction in nursing during the COVID-19 pandemic; 2023 [27]	Study sample: 334 nurses. Variables associated with job satisfaction: position held, gender. Job satisfaction instrument: Job Satisfaction Scale (JSS)—Brazilian version, consisting of 9 domains, rated using a 6-point Likert scale. A total score of 96–108 points indicates dissatisfaction, 109–143 points indicates indecision, and 144–172 points indicates job satisfaction. Country: Brazil.	Among the nurses surveyed, 0.3% reported dissatisfaction with their professional work, 72.2% expressed mixed opinions about job satisfaction, and 27% were satisfied with their work. Nurses in leadership positions in the domains of remuneration (*p* = 0.003), promotion (*p* < 0.001), and operational conditions (*p* = 0.028) demonstrated higher job satisfaction. No statistically significant differences in mean job satisfaction domain scores were observed by gender (*p* > 0.05). CROSS: 34/40.
15	Savitsky B, Radomislensky I, Hendel T; Nurses’ job satisfaction during COVID-19 pandemic; 2021 [28]	Study sample: 133 nurses. Variables associated with job satisfaction: workplace, position, intention to leave the profession, limited availability of personal protective equipment, working with patients with COVID-19. Job satisfaction instrument: Job Satisfaction Questionnaire containing 28 questions (4 questions from the Minnesota Satisfaction Questionnaire, 17 items from the Measure of Job Satisfaction tool, 3 items identical to the MSQ and MJS, and 4 additional original questions). Items were rated on a 5-point Likert scale, with a maximum possible score of 140 points. Higher scores indicate higher job satisfaction. Country: Israel.	The mean job satisfaction score among nurses was 3.6 (SD = 0.6). Community nurses demonstrated higher job satisfaction (*p* = 0.008) than nurses working in hospital wards. Holding a managerial position was associated with higher job satisfaction (*p* = 0.006). Nursing staff who planned to leave the profession had lower job satisfaction (*p* = 0.011). Limited availability of personal protective equipment (*p* = 0.039) and working with patients infected with SARS-CoV-2 (*p* = 0.013) were also associated with lower job satisfaction. CROSS: 34/40.

Source: Authors’ own elaboration based on studies included in the systematic review. Note: Statistically significant findings are explicitly identified and accompanied by the corresponding *p*-values, where reported in the original studies. Non-significant findings are indicated separately. Descriptive findings, for which statistical significance was not reported, are presented as descriptive observations and should not be interpreted as evidence of statistically significant associations.

## Data Availability

No new data were created or analyzed in this study. Data sharing is not applicable to this article.

## Supplementary Materials

The following supporting information can be downloaded at: https://www.mdpi.com/article/10.3390/healthcare14213817/s1.
