# Supplementary material for: Job Satisfaction Among Healthcare Professionals During and Directly After the COVID-19 Pandemic: A Systematic Review of Psychosocial, Organizational and System-Level Factors
Source: Healthcare (Basel). 2026 Nov 9;14(21):3817. doi: 10.3390/healthcare14213817 (PMC13762566; doi:10.3390/healthcare14213817)
Supplement: Supplementary file 1 [file healthcare-14-03817-s001.zip › healthcare-4633746-supplementary.pdf]

PRISMA 2020 Checklist

Manuscript: Job Satisfaction among Healthcare Professionals During the COVID-19 Pandemic: A Systematic Review of Psychosocial, Organizational and System-Level Determinants.

No.	PRISMA 2020 item	Assessment	Location / basis in manuscript
1	Title: identification of the report as a systematic review	Met	Title; p. 1
2	Abstract: structured summary consistent with PRISMA for Abstracts	Met	Abstract; p.1
3	Rationale for the review in the context of existing knowledge	Met	Introduction; pp. 1–3
4	Objectives or review questions	Met	Introduction; p. 3
5	Eligibility criteria and how studies were grouped for the syntheses	Met	Sections 2.2 and 2.7; pp. 3–4
6	Information sources and date of the last search for each source	Met	Section 2.3; p. 3
7	Full search strategies for all databases and limits used	Met	Section 2.3; p. 3
8	Selection process for records and reports, including number of reviewers and independence	Met	Section 2.4; pp. 3–4
9	Data collection process, including number of reviewers and independence	Met	Section 2.4; pp. 3–4
10a	Outcomes and other variables sought, including definitions and assumptions	Met	Sections 2.2 and 2.7; Table 2
10b	Assumptions about missing or unclear data	Met	Sections 2.4 and 2.7
11	Methods used to assess risk of bias / methodological quality of included studies	Met	Section 2.6; Table 1; pp. 4–6
12	Effect measures used to present or synthesise results	Met	Sections 2.7 and 3.2; Table 2

No.	PRISMA 2020 item	Assessment	Location / basis in manuscript
13a	Process used to decide which studies were eligible for each synthesis	Met	Sections 2.7 and 3.2; Table 2
13b	Methods used to prepare data for presentation or synthesis	Met	Section 2.7; Table 2
13c	Methods used to tabulate or visually display results of individual studies and syntheses	Met	Table 2; Sections 3.2–4; pp. 5–10
13d	Methods used to synthesise results and rationale for the chosen approach	Met	Section 2.7 and 3.2; Table 2
13e	Methods used to explore possible causes of heterogeneity	Not applicable	Sections 3.2 and 4.4
13f	Sensitivity analyses	Not applicable	Not described; no meta-analysis reported
14	Methods used to assess risk of bias due to missing results (publication bias)	Not applicable	Not described
15	Methods used to assess certainty or confidence in the body of evidence	Not assessed	No formal assessment of certainty or confidence in the body of evidence was conducted.
16a	Results of the search and selection process, including numbers at each stage, preferably in a flow diagram	Met	Section 3.1; Figure 1; p. 5
16b	Reports retrieved, assessed for eligibility, and excluded, with reasons for exclusion	Met	Section 3.1; p. 5
17	Characteristics of each included study	Met	Table 2; pp. 7–10
18	Results of risk-of-bias assessment for each included study	Met	Table 1; p. 6
19	Results of individual studies, including summary statistics and effect estimates/precision where appropriate	Met	Table 2; pp. 7–10
20a	Results of each synthesis, including a brief summary of the characteristics and risk of bias of contributing studies	Met	Sections 3.2–3.3 and Section 4
20b	Results of all statistical syntheses, including estimates and	Not applicable	Section 3.2

No.	PRISMA 2020 item	Assessment	Location / basis in manuscript
	precision		
20c	Results of investigations of heterogeneity	Not applicable	No formal analyses described
20d	Results of sensitivity analyses	Not applicable	No formal analyses described
21	Assessment of risk of bias due to missing results	Not applicable	No results of such an assessment identified
22	Assessment of certainty or confidence in the evidence for each main outcome	Not assessed	No formal assessment of certainty or confidence was conducted for the main outcomes.
23a	Interpretation of the results in the context of other evidence	Met	Discussion; pp. 10–13
23b	Limitations of the evidence included in the review	Met	Sections 4.4; p. 13
23c	Limitations of the review processes	Met	Section 4.4; p. 13
23d	Implications of the results for practice, policy, and future research	Met	Conclusions; Implications for practice and healthcare systems; p. 13,
24a	Registration of the review: registry name and registration number	Met	Section 2.1; p. 3
24b	Where the review protocol or protocol information can be accessed	Met	Section 2.1; p. 3
24c	Amendments to information provided at registration or in the protocol	Not applicable	-
25	Sources of financial or other support and role of funders	Met	Funding; p. 14
26	Competing interests	Met	Conflicts of Interest; p. 14
27	Availability of data, data-collection forms, code, and other materials	Met	Data Availability Statement; p. 14
